# Abstracts of papers presented at the International Symposium on Laboratory Automation and Robotics (October 1992)

**DOI:** 10.1155/S1463924692000397

**Published:** 1992


Journal of Automatic Chemistry, Vol. 14, No. 6 (November–December 1992), pp. 201–222

### Abstracts of papers presented at the International Symposium on Laboratory Automation and Robotics (October 1992)

### Keynote 1: Practical aspects of laboratory automation in pharmaceutical development

*Eugene J. McGonigle, Schering-Plough Research Institute, Kenilworth, NJ*

An early perception of laboratory automation was 'automated analysis' and its focus on the manipulative aspects of analytical chemistry. Automation freed the chemist to carry out other tasks in pharmaceutical analysis. With the preference for HPLC in pharmaceutical analysis, autosamplers and injectors were the earliest and most obvious improvements. This was followed by the advent of computers and user-friendly software to facilitate chromatographic measurements and calculations. Today, robotics, in combination with laboratory automation and data management systems, can effect completely automated analytical methods in which many, if not all, of the physical manipulations require minimal chemist intervention. Broad in scope, modern laboratory automation facilitates scheduling, analysis, data handling and processing, reporting and statistics, as well as project and resource accountability.

The payback from using this technology is obvious. Operator manipulations, variability and error are minimized. Sample throughput and turnover can be optimized. Laboratory productivity can be monitored, thereby increasing credibility. However, nothing is free so there are 'costs' as well. Validation is more extensive and critical. Entire laboratories can be vulnerable to computer malfunction. Eugene McGonigle discussed these considerations with perspective to the practical utilization of laboratory automation in research and development laboratory.

### Keynote 2: Rational drug design, planned serendipity and the place of automation in pharmaceutical lead discovery

*Sean O'Connor, Bristol-Myers Squibb, Pharmaceutical Research Institute, Wallingford, CT*

The two decades from 1970 to 1990 saw a very rapid escalation in the cost of bringing a new prescription drug to market. This, together with political pressure to restrain price increases, the appearance of the first mega-drugs ($1 billion-plus in annual sales) and the perception that all was not well with traditional medicinal chemistry, made the senior management of many pharmaceutical companies extremely susceptible to the allure of 'rational drug design'. Sean O'Connor argued that the development, during this same period, of programmable material handling equipment and its use in high throughput screening, served as a corrective to what, otherwise, was becoming a headlong lemming charge in pursuit of simplistic answers.

### DATA MANAGEMENT

### Validation and comparison of an automated titration system

*Darrell A. Kelly and James D. Cunningham, Monsanto Company, Pensacola, FL*

The analytical results from an automated nonaqueous titration system of the amine end groups on nylon 66 or 6 polymer using phenol/methanol solvent and dilute HCL acid were described. Various studies on three automatic titration systems located at plant sites in Florida and South Carolina were discussed. The manual methods and the automatic methods for each location were compared by paired t tests and found in most cases to be identical. Blind testing assigns the relative contributions to the total variance as 76% from the manufacturing process and 24% from the robotic analytical methods. The third study demonstrated the correlation between the two manufacturing laboratories as measured by a round-robin study and through blind sampling.

### Data versus information in the automated laboratory

*Stephen R. Metzner, Monsanto Agricultural Company, St. Louis, MO*

The need to improve the quality of analytical data in the face of increasing work loads, decreased budgets and fewer personnel has lead to an interest in laboratory automation. This frequently results in a variety of intelligent instruments (some equipped with their own DOS or Unix based computer systems) each producing data in a format incompatible with everything else.

A laboratory is often forced to use a LIMS system imposed on it by the larger company or MIS group. Often, this LIMS system becomes nothing more than a job scheduler or electronic notebook, requiring extra time to transfer data to it along with increased risk of transcription errors.

Four Zymark robots are part of Analytical Technology's automated systems which served Monsanto's Animal Science Division. Robots also suffer from many of the same problems which plague other intelligent instruments. They are easy to set up as isolated, stand alone units which produce data but not information. The goal of any automated laboratory should be the interchange of information in the form of sample codes (assay number, lost number etc.), sample handling information, raw data and completed calculations with all systems that need this information.

### Integration of independent robotic, chromatographic, and personal-computer system for the analysis of toxicology feed sample under GLP regulations

*Vijay K. Miriyala and John Boyd, American Cyanamid Company, Princeton, NJ*

US Government Good Laboratory Practice (GLP) regulations for toxicology studies require demonstration that test-material levels in diets administered to test animals match those prescribed by the study protocol within a reasonable margin of error. Because of the large number of samples involved for each study and GLP requirements for extensive and detailed documentation of all phases of the process, preparation of treated feed samples for HPLC/GC analysis is a labour-intensive, monotonous, and time-consuming process.

An integrated system which minimizes operator involvement but which produces full, detailed documentation and yet retains the flexibility of a unit-operations approach was developed for sample preparation, chromatographic analysis, data reduction, and report preparation. Sample preparation is carried out by a Zymate laboratory robot which documents each operation on each sample gravimetrically and produces data tables both on paper and on diskette as a DOS file. Chromatographic analysis is accomplished on an independent system consisting of several HPLC and/or GC chromatographs connected to a chromatographic data-collection system provided by Spectra-Physics, which produces DOS files containing the appropriate chromatographical data. Sample-preparation data from the robot and chromatographic data from the data system are imported directly from the respective DOS files into a custom-designed LOTUS 1-2-3 spreadsheet which calculates all results and produces a detailed data table ready for directly inclusion in the final report.

Automation of this analysis has reduced the analyst's hand's-on time involvement to about 1 hour for a run of 30–40 samples. This allows each analyst to handle more studies than was possible previously, in spite of the greatly increased effort required to comply with GLP regulations. In addition, compared to previous manual operations, the system provides increased sample throughput, less variability from analyst, and more precise and accurate results.

### Development of an automated powder distribution laboratory in a pharmaceutical research centre

*P. B. Monnet, V. Thyrault and L. Drugeault, Rhone-Poulenc Rorer, Vitry sur Seine, France*

Powder distribution has become a commonly referred pathway in many research laboratories. Few manufacturers evaluate existing means of distribution, giving technological answers. A pharmaceutical research centre manages a very large quantity of original powders collecting the active matters. Any research project will require some of those specimen (samples), with a quick and accurate delivery. The regular increase of requests for accurate low quantity powder distribution induces automation to support human manipulations.

Distribution and powder data management are not computerized, and most distribution actions are automated. Because of the large variations in the physical characteristics of the powders, two parallel stations, linked by a master personal computer, were used to set up an automated powder distribution laboratory. To prevent contamination and loss, a special endless screw cap was designed to fit the master tube containing the product.

The robotic station and the manual station are similar in terms of global architecture. They are diven by a personal computer including multi-communication card (up to 8 RS232C-V24 ports) and analogic-digital input-output card, and run under an MS-DOS operating system with a multi-task program written in Turbo Pascal. Balances, barcode readers and barcode printers are common peripherals for these stations.

One cylindrical robot on track with five and half axes, three automated storage units, three automated dispensing units, two automated capping units, one automated label-sticking unit are the robotic station specific peripherals. They are mainly self-sufficient, driven by their own programmable-automation. The station computer orders them to act at a precise time, listening to their answers until the end of their task. The direct-current motor of each dispensing units is driven directly by the computer according to a specific algorithm which refers to an expert data base, and physical characteristics of the powders.

---

### LABORATORY WORKSTATIONS

### Preparation of standard solutions: the workstation approach using a Zymate robot

*James R. Ormand, The Dow Chemical Company, Health and Environmental Sciences, Midland, MI*

Many robotic systems are designed to perform operations highly specific to each laboratory. The applications for these systems are dedicated to processing one type of sample with high throughput, and their use is limited to a few users who need this specific type of sample processing. While these systems are valuable in performing these specific tasks, their versatility is rarely used to the fullest potential. To capitalize on this versatility, a more universal workstation type of design would be desired. Aspects of this workstation include flexibility, rapid turnaround, and multiple users.

In the example described, a Zymate robot was used as a workstation to accomplish a nearly universal, repetitive, short-term laboratory task. This application preparation of analytical standard solutions gains its value from accuracy, precision, and convenience of the workstation design.

The application performed trace standard preparation for GC, LC or GC/MS analyses, and use equipment that is common to many robotic systems, (balance, MLS, capper, and syringe hand). Following manual preparation of a stock solution, the program:

(1) Prompts the user for all the necessary information, including the desired solution concentrations.
(2) Performs all calculations needed to produce the end results.
(3) Performs serial dilutions of a stock solution with gravimetric tracking of all dilutions.
(4) Transfers the standard to the autosampler vial of choice.
(5) Provides an audit trail.

James Ormand discussed the design of the application, results from several months of standard preparation, the accuracy and precision of the preparation technique; and future improvements.

### A robotic workstation for the processing of soil samples

*L. M. Ford, H. A. Boll and O. W. Godfrey, Eli Lilly & Company, Lilly Corporate Center, Indianapolis, IN*

The Soil Workstation, consisting of a Mitsubishi Ex Movemaster Robot interfaced with an automatic plate feed and miscellaneous hardware, processes 24 soil samples in one operation. A portion of each soil sample is suspended in a 2 ml aliquot of buffer and sonicated for 90 s. Three ten-fold serial dilutions are made, and 100 μl from the undiluted suspension and from each dilution are delivered onto an agar plate containing 4 copies of 16 different media. During delivery, the plate is rapidly shaken to ensure even dispersal over the surface of the agar. The undiluted soil suspension is placed in a refrigerated rack, and subsequently stored in liquid nitrogen after all 24 samples are processed. A compound that may selectively kill certain micro-organisms is added to another portion of the original soil sample. After overnight incubation at room temperature, these treated soils are processed and plated in the same manner as the untreated samples. When growth has occurred, the mult-media plates are visually inspected to determine the optimal medium and plating dilution, and whether the selective treatment was effective. Either the treated or untreated sample is then delivered onto an agar plate containing the proper medium at the appropriate dilution, and incubated to allow colony growth. The colonies are selected and inoculated into fermentation broth as previously described (ISLAR 91). As an alternative to adding a selective agent for treatment, the system has the capability of filtering soil suspensions, and subjecting the filtrates to either heat shock or ultra-violet irradiation prior to plating.

### Automation of radioanalytical sample preparation

*James W. Dillard, Oak Ridge Laboratory, IT Analytical Services, Kingston, TN*

Sample preparation techniques in radioanalytical laboratories have not kept pace with advances in radiocounting technologies. With recent advances in robotics and microwave heating technology, new sample preparation techniques can significantly improve quality, productivity and laboratory safety. Commercial radioanalytical laboratories, that use labour-intensive sample preparation methodologies will realize a significant competitive edge in the future high-tech laboratory market by implementing robotics technologies.

Laboratory robotics now allow many previously labour-intensive sample preparation methodologies to be automated. A laboratory robotics system can now be installed as a laboratory workstation that can perform such tasks as blending, weighing, pipetting, bulk liquid transfer, liquid-liquid extractions, column chromatography, evaporation, etc. When combined in the appropriate sequence and controlled by computer, most steps of a radioanalytical sample preparation procedure can be performed without human intervention.

A recent commercially available robotics open vessel microwave digestion system is an excellent example of the successful matching of robotics manipulation and microwave heating of a reaction vessel. The Oak Ridge Laboratory has successfully utilized this system in dissolution of samples for radioanalytical analysis. This totally automated system has reduced a laborious several shift operation to a few hour process per sample.

James Dillard discussed the technical aspects of open vessel microwave digestion; the application of robotics workstation; and the equipment/computer interface for a totally automated system.

---

### VALIDATION

### Validating a bioanalytical robotic system: developing system reliability and conducting a formal validation

*Julie J. Tomlinson, Clinical Pharmacology, Glaxo Inc., Research Triangle Park, NC*

Bioanalytical robotic systems are routinely used in Julie Tomlinson's laboratories to meet the demands of the drug development process. To reliably validate automated assays and meet (or exceed) study deadlines, the benefits of the systems are taken advantage of and the limitations avoided. For example, the laboratories:

(1) Select bioanalytical techniques that are well-suited to robotic software and hardware (i.e. are robot-friendly).
(2) Use gravimetric feedback loops during sample processing that ensure that all liquid transfers are precise and accurate.
(3) Develop feedback mechanisms between the robots, HPLCs, data systems and other components of the robots' environments.
(4) Modify hardware and software to address specific chemistry/system incompatibilities that arise during continuous system operation.
(5) Write software routines that permit inexperienced users to easily restart serialized runs when the inevitable system failures do occur.

Once a robot system is running without failure, the systems and accompanying analytical methods are validated to meet specific, rigorous standards of performance. Bioanalytical validation criteria for the pharmaceutical industry have recently been established by North American and European regulatory agencies. These criteria have been added to standard operating procedures for method validations. The procedures for validating robotic systems using those criteria were described.

### Conversion of a manual solid phase extraction procedure to the BenchMate Workstation and to the HPSPE PySection

*Mark E. Arnold, Stephen J. Dennell, Eugene Ivashkiv and Allen I. Cohen, Bristol-Myers Squibb Pharmaceutical Research Institute, New Brunswick, NJ*

The manual solid phase extraction procedure for BMS181168 and its metabolites in plasma was automated on two extraction systems: the BenchMate and the High Performance Solid Phase Extraction (HPSPE) PySection on an Zymate XP robot. The extraction procedure uses barrel-style cyclohexyl SPE columns containing 200 mg packaging. Manually, samples are extracted, dried, reconstituted in mobile phase and placed into an HPLC autosampler. The HPLC injects one-half the reconstitution volume into a 15 cm C18 column with UV effluent detection at 254 nm. The labour intensive sample preparation was automated on a Zymark BenchMate. The BenchMate is capable of performing the extraction with a single sample extraction time of 9 min. Extracts were dried in a TurboVap Concentration Workstation, reconstituted in mobile phase and placed into an HPLC autosampler. To further reduce active personnel involvement, the extraction was placed on a Zymark XP robotic system using a beta-test HPSPE station. All programming of the HPSPE station was in PyTechnology EasyLab commands. All sample preparation steps, including HPLC injection were performed by the robot. Minor modifications to the extraction method were required for each progressive enhancement in automation. Comparisons of the three methods were made. Results of each validation were presented and the results of the BenchMate workstation and HPSPE station were compared.

### Validation of a robotic dissolution assay for an immediate release/sustained release tablet

*Maria E. Guazzaroni, Pfizer Inc., Brooklyn, NY*

The USP dissolution method for a tablet with an immediate release and a sustained release components was validated for the Zymate XP dissolution robot. The procedure consisted of USP dissoluton followed by HPLC analysis. Acceptable linearity, reproducibility and recoveries were established for two active ingredients. The robotic and manual methods were also compared.

### A unified approach to validation of multiple robotic systems in a stability laboratory

*S. W. Swieck, Jr., J. R. Tricome, P. M. Chambers and N. Muhammed, Bristol-Myers Squibb Company, Syracuse, NY*

Several Zymark automated robotic systems are now in operation processing various pharmaceuticals in the authors' laboratory. These units are operated by various analysts, initiating specific robotic procedures using validated HPLC methods in the evaluation of individual compounds.

A practical validation procedure has been developed to ensure compliance with GMP regulations. In conjunction with the validation procudure, a system for creation, transfer, and implementation of robotic programming was also developed to assure proper functioning of the robotic systems. With the unified validation approach, operation of the robotic systems by robotic users, utilizing various robotic programs, will comply with FDA/GMP regulation.

### Considered in the validation of a robotic method for content uniformity of a tablet dosage form

*Stephen Scypinski, Theodore Sadlowski, and Peter F. George, Pharmaceutical Analysis Research & Development, Hoffmann-La Roche Inc., Nutley, NJ*

The use of robotics and laboratory automation in pharmaceutical analysis is well-documented and established. The Zymark PyTechnology system is used by the authors to enhance productivity by analysing samples of drug products in Phase III clinical trials. Typically, a project which has reached this stage of drug development has associated with it a large number of samples, including those attributable to clinical release, manufacturing scale-up, process validation and stability.

A robotic method used for such analyses must meet the established cGMP criteria used to validate its manual counterpart. These criteria consist of the usual validation parameters, such as precision, accuracy and recovery. However, the validation of the robotic method must extend to calibration operations such as the pipetting and transferring of liquids. In addition, the analyst must endeavour to avoid making major changes to the body of the method as it is adapted to the robot, to ensure that the procedural steps of both methods remain similar.

This presentation discussed some of the authors' experiences, frustrations and, most importantly, successes in the transfer of a manual method for tablet content uniformity to a robotic system. The validation of this procedure and its comparison with the manual method, along with the resulting data, were highlighted.

### Validation of a tablet processing workstation

*K. R. Lung, J. S. Green, P. K. Hovsepian and J. A. Short, The Du Pont Merck Pharmaceutical Company, Experimental Station, Wilmington, DE*

Although laboratory robots have been used for the analysis of pharmaceutical dosage forms since 1984, interest in GMP compliance for laboratory robots did not appear until recently. Based on the authors' previous experience in validating robotic devices used in the blood-banking industry, a validation scheme for the Tablet Processing Workstation was designed and implemented.

---

### MANAGING LABORATORY AUTOMATION

### Laboratory automation: changing the role of the technical professional

*Kevin J. Halloran, Wallace Laboratories, Carter-Wallace Inc., Cranbury, NJ*

Innovation and research go hand-in-hand. After all, the research laboratory is the birthplace of technical innovation. Each year new scientific techniques are discovered, and new forms of instrumentation are developed. The technical professional (chemist, physicist, pharmacist etc.) is accustomed to the evolutionary nature of his or her environment. These scientists continually update their knowledge of their field in order to best apply these newly developed scientific techniques.

During the past 10 years, however, different types of innovation have been developed that do not necessarily enhance the scientific abilities of the laboratory. These innovations, in the form of laboratory robots, intelligent instruments and sample processing workstations, were designed to increase laboratory productivity and decrease operating cost. Coupled with a sophisticated laboratory information management couputer system, this equipment should significantly reduce the amount of manual labour generated in the laboratory.

In this presentation, Kevin Halloran explored how the spread of laboratory automation can affect the working environment of the research laboratory. The discussion was concentrated on the analytic chemistry laboratory in the pharmaceutical industry.

### Managing the design and implementation of an agrochemical central sample preparation laboratory

*Alan N. Mannherz, American Cyanamid, Agricultural Research Division, Princeton, NJ*

In order to provide a more efficient and centralized means of chemical sample preparation for primary *in vivo* and *in vitro* biological screening, a new robotic system was designed and implemented. The existing manual operation had been executed repetitively by several biologists every week for almost 30 years. The conversion of that operation into an automated process was a time-consuming, tedious process of meetings, discussions, and decision-making.

Sparked by a suggestion from one of the testing groups, a committee was formed to organized relevant screening data (testing schedules, sample weights, solvents, etc.); identify the differences or conformaties existing between screening groups; and design a workable system of automating all steps of the manual operation. The most desirable concept was to design a system that was based on volumetric rather than gravimetric principles, which would allow samples to be prepared without precise weightings. This concept seemed amenable to the use of robotics. The management and implementation of the new system was given to the 'Chemical Library' which had the responsibility for the acquisition, registration, storage, and retrieval of screening compounds and their data.

Several of the key issues which had to be addressed included the following: Who are the current internal and external users of robotics and automation? Can all aspects of the manual system really be duplicated? What automation hardware and software should be purchased? How should insoluble samples be handled? Can we prevent contamination between samples? What computer systems are necessary? What laboratory facilities are needed? How should the operation be staffed? Vendor selection was also an important consideration. Which vendor of robotic and automated systems would provide us with the technical and engineering advice; create a user-friendly, workable system; and be able to fully support that system once it was installed?

The trials, tribulations, and successes of designing and implementing a new robotic system from scratch were presented. The transition from a time-consuming, individualized manual system of compound preparation to a fine-tuned, centralized robotic operation were discussed.

---

### AUTOMATING PHARMACEUTICAL METHODS

### Robotic content uniformity testing of a family of effervescent tablets

*Paul J. Barrett, Miles Inc. Elkhart, IN*

The introduction of content uniformity testing in the Quality Assurance Laboratory at Miles Inc. meant a 10-fold increase in the number of samples to be tested. While a stop-gap procedure using manual preparation techniques was underway, a Zymate robotic system was set up and validated in order to automate the tablet preparation for HPLC analysis.

The robot meant that the work did not suffer. It alone generated over 18 000 valid analytical results in four months; in part, because it is able to combine three separate manual procedures into one preparation for the quantitation of five ingredients. This robot features the use of four different types of disposables and is restricted to analysing only one product at a time.

A later second robot was given major design changes that eliminated any need for disposables. It utilizes data handling software which allows information to be automatically transferred from Beckman CALS to the robot for the appropriate processing of multiple products. Extensive error checking on weights and processing times is included with this system.

The latest robot design features a simplified liquid handling module in order to minimize unwanted sample dilution and carry-over.

### Automation of the rate-limiting steps for high capacity random screening: compound weighing and dissolution

*Mary K. Murdoch, Mary E. Russell, Naved Surve, Xiaowen Ma, Melissa S. Drelich, Raymond P. Smith, Honora Lukas, Christopher Duston, Andrew Hanson, James R. Paterniti, Jr., David B. Weinstein and Dennis S. France, Department of Atherosclerosis and Vascular Biology, Sandoz Research Institute, East Hanover, NJ*

Efficient compound screening against enzyme, receptor, and other biologically relevant targets can play an important role in discovering pharmacologically novel leads for drug development. Sandoz has successfully exploited an extensive library of natural compounds (for example fungal broths). However, the authors' collection of pure chemical compounds remain largely unexplored. The chemical substance collection at the Sandoz Research Institute, for example, contains more than 25 000 compounds of which more than 75% are available in quantities greater than 300 mg. It would require two persons working full-time over three years, to the exclusion of all other duties, to weigh and dissolve these compounds with current standard operating procedures. To accelerate this process and to circumvent the inherent laborious and tedious nature of compound weighing, a robotic workstation, the Zymark BenchMate (BM), has been adapted to the task of automatic weighing and dissolution. During each cycle, the BM tares 200 test-tubes (16 × 100 mm) and stores the weights until needed. A team of two or three persons then non-quantitatively transfer an approximate amount (optimal range: 0·5 to 10 mg) of each compound into the tubes, a task which typically requires 1·5 h. The tubes are returned to the BM where they are reweighed. Dimethyl sulfoxide (DMSO) is delivered to the tubes to a target concentration of 2·0 mg/ml of compound and the tubes are briefly vortexed. An equal volume of water is then delivered to yield a final concentration of 1·0 mg/ml of compound and 50% DMSO. Following completion of this unattended robotic run (4 h), the electronic record of the actual weights of the compound can be downloaded into an ORACLE database which then automatically updates the inventory of each compound in the local inventory (PIDMS) database. To demonstrate the practicality of this approach more than 6000 compounds have been weighed and dissolved (mean ± sem; 4·83 ± 4·0 mg), largely from our chemical intermediate collection. The chemicals are stored at 4 °C both in original tubes as well as in a 96-well microtiter tube format which is convenient for both manual and automated pipetting and dilution devices. Time-budget analysis reveals that one person hour is required for the processing of 60 compounds. This represents at least a 10-fold improvement over manual methods. In summary, automated access to the local Sandoz library of pure chemical compounds should enhance lead-finding screening programmes for a variety of therapeutic targets.

### The use of a Zymark tablet processing workstation for the analysis of pharmaceutical tablets

*Ligaya G. Lagade, Cheryl A. Cupo, Taranjeet K. Sawhney, Dorothy E. Martynuk, Kevin J. Halloran and Henry J. Mortko, Carter-Wallace Inc. Cranbury, NJ.*

Content uniformity analysis is one of the most common labour intensive analyses performed by the analytical support laboratory. It requires a minimum of 10 individual tablet assays per batch. This type of high repetitive analysis is a logical choice for automation.

The tablet processing workstation is the latest addition to the series of automation products introduced by Zymark Corporation. The TPW, as it is often called, was designed to perform whole tablet assay. The combination of a rugged content uniformity method with a TPW is a perfect match of sample analysis with automation.

The manual HPLC potency assay and content uniformity analysis procedures for a pharmaceutical product have been adapted and validated for sample preparation by a Zymark Tablet Processing Workstation. The procedures were adapted to meet the demand for a quick turn-around time for content uniformity analyses of process validation samples.

In the paper, the studies that were performed to validate the TPW procedure were discussed. Additonally, the impact the TPW has made on meeting deadlines for the large number of process validation sample analyses was explained.

### Automated screening of enzyme inhibitors

*Richard Harrison, Merck and Company, Rahway, NJ*

The modern enzymology laboratory places a large effort in the screening of natural products and medicinal chemistry compounds. The goal being to identify specific, potent enzyme inhibitors which will serve as lead compounds in the drug discovery process. As many as 10 000 different compounds must be evaluated before a suitable candidate is located, therefore an efficient means of automating this process is essential.

Richard Harrison and his colleagues have developed a method to allow high sample throughput for several enzyme screens. Automation has been achieved by the use of a robotic HPLC autosampler interfaced to a conventional HPLC system. The instrument titrates inhibitor, adds enzyme and substrates at precisely time intervals, quenches reactions, and injects solutions onto the column. Customized software allows unattended data reduction and analysis. Reaction temperature control, and vortex mixing are all achieved using a custom designed rack. The entire operation is automated, allowing throughput of 10 samples/instrument/day. This increases throughput 200% when compared to the same procedure performed manually.

---

### PHARMACEUTICAL RESEARCH

### Flexible and fast automated HPLC assays for evaluating drug metabolism and pharmacokinetics

*John Franc, Bristol-Myers Squibb Company, Syracuse, NY*

As pharmaceutical companies endeavour to shorten the time to market and maintain the quality of a portfolio of new products, laboratory automation has increasingly taken on a strategic role as a means for providing fast, flexible and accurate chemical analyses of new drug entities. Automated analytical systems – consisting of robotics and *people* – must be sufficiently flexible to accommodate a wide range of extraction techniques, in light of the differences in drug/drug metabolite chemical nature (polarity, acidity/basicity) that often exist. Moreover, the key elements of these systems must be prepared to perform more analyses with less head count than before, since the goal among the leading drug companies now is to contain R&D costs. These facts pose serious challenges for automated analytical systems, in terms of improving flexibility and reducing setup times. The objectives then of any automated analytical system are to provide data to support drug development that is both timely and meaningful, as well as provide the means to increase throughput and minimize the outsourcing costs associated with contract labs.

A fully automated liquid-liquid extraction/HPLC procedure was developed and validated for nefazodone and its metabolites, m-chlorophenypiperazine (mCPP), hydroxynefazodone (HO-NEF), and para-hydroxynefazodone (p-HO-NEF) in human plasma. Subsequent to assay validation, over 18 000 samples from clincial and non-clinical studies were analysed by this method, resulting in the timely NDA filing for nefazodone. The increased analytical capacity provided by this automated procedure during 1990 and 1991 enabled the performance of these analyses in-house, thereby substantially reducing contract lab costs.

Since the development of the first automated procedure, two other HPLC procedures have been developed and validated in response to the immediate need (<1 month) for additional plasma concentration data for the major nefazodone metabolite, M4. First, the original automated liquid-liquid extraction procedure was modified and validated to use an acidic buffer ($NH_4OAc$-pH6) to enhance hydrophobic interactions and thus enable the recovery (44%) and quantitation of M4. Then an automated solid phase extraction (SPE) procedure was developed and validated using a weak buffer (0·01M $Na_2HPO_4$–pH7) and a Cyclohexyl BondElut column to enable both hydrophobic and ionic interactions. The SPE technique resulted in high recoveries (90–95%) of NEF, M4, mCPP and HO-NEF, and has subsequently been used to support nefazodone studies, worldwide.

### Improved sample prepartion methodology by interfacing a Hamilton MPH2200 liquid handling station with a Zymark robot

*Joseph J. Yacobucci, Jeffrey Guss and Derek J. Hook, Bristol-Myers Squibb, Pharmaceutical Research Institute, Wallingford, CT*

In order to extend the unattended running time of a Hamilton MPH22000 liquid handling station, a Zymark robot was added to replace consumables and remove completed microplates. This system supplies several of the authors' screening laboratories with fermentation extracts aliquoted in screen specific arrays.

The open programming environments of the Hamilton station and Zymark robot system allows considerable flexibility in developing sample preparation methods that are adaptable to the demands of different screens. This system is more user-friendly than competing systems and is not constrained by a hardware or software specific robot manipulator.

### High volume screening: challenges facing sample delivery and proprietary information managers

*John Brussolo, Parke Davis Pharmaceutical Research Division, Ann Arbor, MI*

High volume screening has become a significant part of drug discovery. The large number of samples required by the high volume assays and the data generated by them present new challenges for sample delivery staff and proprietary information managers. The Chemical Biological Information (CBI) Department has developed an automated sample preparation and information handling system. CBI has a Beckman Instruments robot that prepares dissolved samples (estimated weight) in 96-well microtiter format for qualitative testing purposes and a Zymark robot that prepares dry or dissolved samples (accurate weight) for quantitative testing purposes. The information associated with the sample pre- and post-experiment can be transferred electronically among several computers. All proprietary information is stored in relational database tables on Digital Equipment Corporation VAX mainframe computer.

### Modulation of gene transcription: a fully automated cell based, high throughput drug screen

*Colin Goddard, Rich Michitsch, Keith Riccardi and Ralph Infantino, Oncogene Science Inc., Uniondale, NY*

Oncogene Science Inc. has spent the last four years pioneering a drug discovery strategy which targets tissue specific regulation of gene transcription as a means of identifying a novel class of pharmaceuticals. Stable reporter cell lines are constructed which contain 5' regulatory sequences of the gene of interest fused to a reporter gene, the firefly luciferase gene. Cells are grown in the presence of test agents to identify those compounds which specifically up or down-regulate the target promoter as measured by changes in light production from the luciferase/luciferin reporter system. It was envisaged that very high throughput drug screening would be necessary to identify suitable lead compounds, and we immediately committed to fully automating this process. Working in collaboration with Zymark Corporation (Hopkinton, MA), a system has been developed which has the capacity to run in excess of 125 000 cell-based reporter assays per week. Two Zymark XP robots are integrated with a Tecan RSP and custom designed tissue culture incubators on a servo-motor driven turntable to create the core of the system. One end of the system features interchangeable units to facilitate sample prepartion and loading and the other end a series of units for microplate handling in an assay loop. Most units contain modifications which are specific to this system. Cells are grown in the incubators in 96-well plates and shuttled to the Tecan for the additon of drugs and controls. After incubation with drug, the plates are moved through an assay loop at the end of which the light emitted from each well is determined in a microplate luminometer. Data is automatically dumped to a dedicated PC network where it is captured and analysed. A series of QC parameters are run on each plate proper to databasing. Most of the software for this system has been developed at Oncogene and has been developed to maximize flexiblity in terms of the number of cell lines which can be screened concurrently, the number of replicates and controls required and the nature of the assay. Fully integrated automation on this scale offers huge advantages in terms of cost, throughput and accuracy but requires significant development time and in-house expertise. Oncogene Science has three of these systems currently in place and has formed collaborative ventures with several pharmaceutical companies to further exploit this technology.

### Automation of eicosanoid scintillation proximity assays

*S. Paulson, J. Gales, G. Freeman, R. Brooks and M. Milton, Searle Research & Development Division of G. D. Searle & Company Skokie, IL*

A scintillation proximity assay (SPA) for Leukotriene B4 (LTB4) has been developed. SPA is new technology for performing radiommunoassays (RIAs). SPA beads are fluomicrospheres with anti-rabbit IgG or protein A attached. In the SPA procedure, antibody bound radiolabelled ligand which binds to anti-rabbit beta-scintillation counter. Contrariwise, free radiolabelled ligand which is not within close proximity to the fluomicropheres does not emit light. SPA eliminates the need for separation of bound from free ligand.

In the present study, an Amersham LTB4 SPA kit was modified to a microtiter plate format for automation on the Biomek automatic pipetting station and quantitation by a Microtiter Plate Scintillation Counter (LKB Pharmacia Wallac 1450 MicroBeta LSC). The Amersham LTB4 SPA kit protocol was followed with modifications in reagent volumes and incubation times. Four quality control (QC) pools of 4, 8, 16 and 30 ng/ml were prepared and assayed; analytical recovery (%AR) and coefficient of variation (%CV) were determined. The interassay CVs for the 4, 8, 16 and 30 ng/ml QC pools were 10·9% 5·2%, 4·1% and 3·5%, respectively. Interassay ARs for the respective QC pools were 104·8%, 110·5%, 125·6% and 113%. The interassay CVs for the 4, 8, 16 and 30 ng/ml QC pools were 13·9%, 17·4%, 12·8% and 11·8%, respectively. Interassay ARs for the respective QC pods were 88·0%, 87·3%, 102·5% and 97·3%. Turn around time for the LTB4 assay was reduced from three days to 24 hours. Sample throughput increased from 166 samples/run to 330 samples/run. Conversion of conventional RIAs to SPA and adaptation of SPA to a microtiter plate format provides a rapid means of quantifying endogenous compounds using the pharmaceutical evaluations. This technology could be quite easily adapted to a fully automated robotic system in which certain parameters could be programmed for optimization of each individual SPA.

### Automation of molecular pharmacology: principles and pitfalls

*Melvin Reichman, G. D. Searle & Company, Skokie, IL*

The development of radioligand binding assays in the late 1960s and early 1970s allowed measurement of the primary recognition event which initiates stimulus secretion coupling. Today, radioligand binding assays offer an indispensable tool for intelligent drug design and understanding drug actions; however, 'grinding and binding' provides only limited information. On the other hand, the appropriate functional assays *in vitro* can provide a great deal more information on the pharamcodynamic characteristics of drugs (i.e. their intrinsic efficacy). Today, it is possible to automate many aspects of discovery research, including radioligand binding assays, 'signal transduction' assays and smooth muscle assays. Two paths may be taken to realize this end. One involves designing and implementing a robotics system to carry out the specific task which comprise the assay. While this approach offers great capacity in turnover of compounds, it usually takes quite a while to implement and debug the final result. Moreover, dedicated experts apart from the biology involved are usually needed to maintain the 'system approach'. The other approach involves chaining off-the-shelf equipment. While this 'modular (generic) approach'

does not at first yield a fully automated system, it nonetheless can yield a true 'turnkey' facility. In many cases, the lower cost of implementation and maintenance of such a semi-automated, vendor-supported, facility will offset the loses in compound turnover. Melvin Reichman focused on the biological and mechanistic principles and pitfalls involved in automation of discovery research.

---

### ENVIRONMENTAL

### Automated water methanol/isooctane extraction off PCBs from dry soils using laboratory robotics

*Andreas P. Mueller, Bonneville Power Administration, Vancouver, WA*

A completely automated process for extracting PCBs from dry soils was described. The Bonneville Power Administration analyses nearly 5000 soil samples a year for PCBs from its substations throughout the entire Pacific Northwest. Soxhlet extraction of these samples is time consuming, generates a considerable amount of waste, and is only required during verification of clean up after a PCB spill. Since most samples are not for clean up verification, a method was employed from the US EPA Region I Laboratory to save time and solvents. This approach allows the majority of soils to be prepared for analysis with acceptable accuracy and precision. It uses the three solvent system (water/methanol/isooctane) and mechanical agitation to extract PCBs from dried soil samples.

With an acceptable PCB soil extration method in use, and the number of samples increasing yearly, the need arose to automate the procedure. This was accomplished by employing a laboratory automation system with robotics, After a PCB soil sample is submitted to the laboratory for analysis, it is dried in a low temperature oven, crushed, sieved, and loaded onto the automation system. The robot weighs the sample and adds the three solvents. The sample is then extracted using a vortex mixer, and centrifuged to separate layers. A one to ten dilution is performed and the solution cleaned by vortex mixing over an acid. After centrifuging, one ml of the top isooctane extract layer is sealed in a vial for analysis on a GC. Preparation of 40 samples takes less than 11 h.

Initial estimates of time saved by using automation with robotics are 275 technician hours per year, based on a sample load of 4000 soils. The automated three-solvent shake preparation versus Soxhlet extraction doubles throughput and reduces solvent use by 64%.

### Review of solid phase extraction method development/validation work at the Denver Water Department QC Laboratory

*Bruce Hale, Denver Board of Water Commissioners 1600 West 12th Avenue, Denver, CO*

The Denver Water Department (DWD) Quality Control Laboratory has several years of experience with Solid Phase Extraction (SPE) sample preparation techniques. During this time, the Laboratory has looked for ways to improve routine SPE preparations, and speed development of new SPE applications for water analyses. Most recently, the Laboratory has investigated use of Zymark's AutoTrace SPE Workstation to help achieve these goals. The equivalency of the automated system was tested against some routine SPE methodologies. Recovery and precision were improved for most of the chlorinated insecticides and nitrogenous herbicides investigated. The DWD will now be extending use of the AutoTrace to additional SPE applications with significance to the water treatment industry.

### Applications of robotics to the extraction, purification and analysis of pesticide residues in environmental samples

*John M. Becker and John F. Culligan, Jr., FMC Corporation, Agricultural Chemical Group, Princeton, NJ*

Analytical studies for pesticide residues in evironmental samples or agricultural crops often involve repetitive and complex analyses of a large number of soil, water, or plant samples. Analytical procedures routinely include solvent extraction of the analytes from the matrix, liquid-liquid partition purification, solid-phase extraction, solvent evaporation and analyte concentration, and gas chromatographic (GC) analysis. Analyses are usually conducted for pesticide residues at the part-per-billion (ppb) and part-per-trillion (ppt) level. These factors make the analytical procedures for these studies ideally sited to robotics. In an effort to produce consistent and reliable results in these prolonged studies, two Zymark robotic systems have been configured and successfully implemented for residue analyses. One system completes the labour-intensive liquid-liquid and sold-phase extraction step to supplement a manual analytical procedure. A second more complex, custom-built Zymark robotic system was developed and used extensively to complete the entire analytical procedure, from extraction solvent dispensing through analyte isolation, to chromatogram generation on the integrated GC. These robotic systems have been employed for the analysis of several insecticides and their metabolite(s) in soil, sediment, water, and plant samples. Average method recoveries, based on laboratory fortified control samples, are generally in the 80 or 90% range with less than 10% standard deviation, depending on the analyte. The goals of producing reliable, consistent residue data at ppb and ppt levels during these extensive studies involving complex matrices have been achieved through these robotic applications.

### Comparison of a robotic system for inorganic sample preparation to manual digestion techniques

*Gary Ward, Debra Hosford and Ike Tabani, Enseco Inc, Denver, CO*

A robotic system was developed in a major environmental analytical laboratory for the routine preparation of water and soil samples for metals analysis by inductively coupled plasma (ICP) spectroscopy and graphite furnace atomic absorption (GFAA) spectrophotometry. Since regulations required a variety of digestion methods for ICP and GFAA for the major Federal environmental programmes (i.e. RCRA, Superfund, drinking water), the robot system was programmed to perform all the required digestion steps. This eliminated the constant training of inorganic preparation personnel in the multitude of metals 'preps', while saving significant analyst time as it frees up chemists to perform other work. The system typically operates 24 hours a day and has yet to cause problems resulting in in re-preps. Data will be presented on the accuracy and precision of the working system determined with standards, reference materials, and real samples as compared to the performance of manual digestion techniques.

### Automated extraction of herbicides from water

*R. L. Pfeiffer, USDA/ARS, Ames, IA*

The extraction of herbicides from water using solid-phase sorbents such as C-18 is well documented in the literature. An automated robotic system utilizing commercially available technology has been developed in the author's laboratory for the extraction of atrazine, metribuzin, alachlor and metolachlor. As many as 25 samples with volumes ranging from 50 mls to 1000 ml are extracted by passing through a 500 mg C-18 cartridge. Analysis is done by mass spectrometry using the selected ion mode. Recoveries are greater than 80% over a concentration range of 0·2 to 100 ppb.

### Automated liquid/solid extraction of drinking water and TCLP leachates

*Pat Redmiles, Versar Laboratories Inc., Springfield, VA*

Automated liquid/solid extraction was tested for use in two methods: the first was a modification of EPA method 525 for drinking water determination of organic compounds using GC/MS for analysis. The second was a method developed for extraction of TCLP leachates for pesticides and herbicides using GC/ECD for analysis. Each method was evaluated by determining method detection limits for target analytes using the analytical method specified. Comparisons were made with current extraction procedures for TCLP leachates. Optimum operation conditions for each method were also developed.

### Automated extraction of total petroleum hydrocarbons in water by solid phase extraction disks

*Rick Schrynemeeckers, Enseco Labs, Houston, TX*

The leaking of underground storage tanks (USTs) of petroleum products such as gasoline, kerosene and diesel fuel have become a serious concern for the environment. The number of reported releases increases daily as many tanks approach the end of their normal life expectancy. The increased number of detected releases and remediation efforts has resulted in a significant increase in requests for the analysis of Total Petroleum Hydrocarbons (TPHs). The nature of UST analyses dictates rapid turn-around for analyses.

While multiple shifts and additional staff may improve productivity, the ultimate answer is improved productivity through automation. The extraction phase of TPH analysis is the most time consuming portion. The use of Solid Phase Extraction (SPE) media as opposed to liquid/liquid or separatory funnel extraction provides improved reproducibility, significant reduction in solvent use and reduced extraction times. SPE of petroleum hydrocarbons not only provides similar detection limits as conventional extraction procedures, but also reduces the loss of the more volatile constituents.

---

### CUSTOM AUTOMATION

### The automated sample retrieval and analysis system

*Edward G. Kanczewski, Oluwole Aloba, Robert Miller, Patrick Teulie, Veronica Casier and Clifford Buffett, Parke-Davis Pharmaceutical Research Division, Warner Lambert Company, Morris Plains, NJ*

Pharmaceutical submissions to the Food and Drug Administration (FDA) require documented evidence that the formulation to be marketed is stable. Typically, the facilities and resources required to provide such information is extensive. Sample storage, retrieval and analysis have been incorporated into one automated system. New stability studies are initiated while still maintaining the integrity of ongoing studies. Minimal human intervention is provided in response to system prompts. Computer controlled operation of this system provides a bar code verified audit trail and sample analysis report.

### Automation for tests of dosage and valve of pharmaceutical aerosol products

*R. J. Engerer, J. C. Egoville, and T. O. Callum, Rhone Poulenc Rorer Central Research, Collegeville, PA*

Automated systems for aerosol testing have been developed for Weight Loss per Actuation, Valve Dose Delivery (VDD), and Unit Spray Content (USC) tests. The reproducible shaking mechanism permits the formulation and valve performance to be easily tested. Critical specifications such as valve stem depression distance were easily tested due to the precise, adjustable, actuation design.

Validation for the weight loss per actuation test included a comparison to manual testing. Robotic testing did not result in cooling of the canisters as occurred with manual testing due to the slow controlled actuation rate. A 4000-fold increase in productivity was achieved with the robotic system. Laboratory-to-laboratory variations were eliminated and precision increased.

The VDD test is a test to ensure that the prescribed dosage is delivered throughout the life of the aerosol canister. Manual testing and operator-to-operator variation was eliminated. A four-fold increase in productivity was achieved.

The USC test determines the ability of an aerosol product to be delivered into the lungs by simulating the process of inhalation with ordinary laboratory glassware and a vacuum pump. The glassware for eight tests are conveniently mounted and pump vacuum lines are individually adjusted for the proper vacuum air flow. A four-fold increase in productivity was gained.

Automated and manual data will be presented for the above three tests for pharmaceutical aerosol products. Data for the metered valve delivery of the required product dosage over the entire contents of the aerosol canister were presented.

### Automated HPLC mobile phase preparation using a customized Zymate system

*Scott Strong, NutraSweet Company, Mt Prospect, IL*

High performance liquid chromatography (HPLC), especially in NutraSweet's laboratories, is one of the most widely used techniques in chemical analysis. As a result, mobile phase preparation has become a very common duty. Unfortunately, this task can consume much of an analyst's valuable time. By automating the mobile phase preparation task, it would allow an analyst more time to spend on more important responsibilities.

A group of analytical chemists from the NutraSweet Company initiated the concept and developed the design of an automated mobile phase preparation unit. Following the company's specifications, the Zymark Corporation constructed a unit using customized Zymate hardware and software. The unit has two main functions: it prepares a concentrated stock solution; and it takes this stock solution and prepares the appropriate mobile phase. Dispensing of reagents and solvents is done gravimetrically. After preparation, the mobile phase passes through a filter into a reservoir where helium sparging removes dissolved gases. Finally, the unit prints a report listing the amounts of reagents and solvent dispensed, and the pH of the final mobile phase. The unit can prepare as many as six different types of mobile phase in an unattended mode.

The topics discussed included the design of the unit using Zymate hardware and software, operation of the unit, batch-to-batch reproducibility studies, and additional applications. Other topics included savings in man hours and reagents.

### Use of robotics for high-throughput natural products screening

*James C. Walsh, American Cyanamid Company, Agricultural Research Division, Princeton, NJ*

The discovery of novel agents for use in agriculture is facilitated by high-throughput screening of chemicals and natural products. A Zymark robotic system equipped with an XP robot arm was designed and installed to rapidly apply natural products to 9 inch by 9 inch bioassay plates containing agar seeded with an indicator organism. Improved sample volume capacity over spotting or disk techniques was obtained by using 5 mm wells prepared with a proprietary agar welling device built in-house. The wells are arranged in a 12 × 12 matrix compatible with standard microplate footprints.

Fermentation broths are supplied in 24 well microtiter plates and can be directly manipulated using standard microplate fingers. Sample distribution is carried out using a custom 24-channel pipette hand that can simultaneously transfer sample from all 24 wells to the corresponding test wells of an assay plate. Wide bore, 250 microliter pipet tips are used to prevent tip clogging which can occur when sampling fermentation broths. Table space is conserved by incorporation of unique drawer-like agar plate and sample storage shelves which allow vertical stacking and complete access to all plates.

Overall, screening capacity has increased significantly by using robotics compared to manual sample transfer methods. The Zymark system described can distribute 1008 samples to a maximum of 4 bioassay plates (4032 assays) in 3·5 h. Correlation of automated results with manual methods has been excellent. It is now possible to evaluate a larger number of natural products in a greater variety of assay systems over a fixed period of time.

### Automation of sedimentation analysis for aspartame

*Rick Seigler, Ed Ryan, Lito Castelo, Rod Ledford, Kerry Kenny, Laura Northcraft and Joe Saxon, NutraSweet Company, Augusta, GA; and James D. Cunningham, Monsanto Company, Pensacola, FL*

The Augusta NutraSweet manufacturing plant automated the aspartame sedimentation analysis using a Zymate XP robot and custom stations by the authors and Zymark.

This analysis looks for insolubles by dissolving a measured amount of product in a dilute solution of sodium hydroxide and water. The solution is filtered through a filter pad, dried and visually inspected.

50 g of sample are placed in a Gelman filter assembly in a rack. The robot arm picks up the assembly and transfers it to the filtration station where a cover drops down to lock it down. Pneumatically-driven syringes mix in 16 ml of 50% sodium hydroxide and 284 ml of deionized water, a small propeller spins to dissolve the mix, and after a minute, another 500 ml of deionized water is pumped into the solution.

Vacuum is switched on at the base of the station and filtration of the solution is monitored by an capacitive sensor probe in the waste line. Filtration continues until the value corresponding to a dry line is returned. A spray nozzle supplied by a small peristaltic pump drops down to wash the funnel and capacitance is measured again to signal filtration completion.

To dry the pad, the arm moves the assembly over to another station where a cover drops down and a hot air flow is fed in through the top. A vacuum pump connected to the base pulls the hot air through the pad for a timed cycle and residual moisture is removed. The arm returns the assembly to the rack where the pad is evaluated by the operator.

The advantages of going to an automated system involve mainly increased throughput of samples with the ability to run samples concurrently, washing of glassware eliminated, decreased variation from analyst to analyst, and liberation of laboratory personnel for more demanding tasks.

### Peristaltic pumps for waste disposal

*G. W. Griffith, Allied-Signal Inc., Kansas City Division, Kansas City, MO*

Laboratory robots are capable of generating large volumes of hazardous liquid wastes when used to perform chemical analyses of metal finishing solutions. A robot at Allied-Signal Inc., Kansas City Division generates 30 gallons of acid waste each month. This waste contains mineral acids, heavy metals, metal fluorides, and other materials. The waste must be contained in special drums that are closed to the atmosphere. The intial disposal method was to have the robot pour the waste into a collecting funnel, which contained a liquid-sensing valve to admit the waste into the drum. Spills were inevitable, splashing occurred, and the special valve often didn't work well. The device also occupied a large amount of premium bench space.

Peristaltic pumps are made to handle hazardous liquids quickly and efficiently. A variable-speed pump, equipped with a quick-loading pump head, was mounted below the robot bench near the waste barrel. The pump inlet tube was mounted above the bench within easy reach of the robot, while the outlet tube was connected directly to the barrel. During operation, the robot brings the waste liquid up to the pump inlet tube and activates the pump. When the waste has been removed, the pump stops. The procedure is quick, simple, inexpensive, safe and reliable.

---

### CHEMICAL ANALYSIS

### Automation of nonaqueous titration analysis system

*James D. Cunningham and Darrell A. Kelly, Monsanto Company, Pensacola, FL*

An amine-end analysis system was automated using a Zymate robot, purchased peripheral equipment, and some equipment designed by the authors. The technician manually weighs the sample and transfers the weight electronically to the PC. The technician must log in the samples and inform the system to PREPARE the samples. After these technician interactions, the automatic system takes the weighed sample, adds methanol, phenol and places the sample on a magnetic stirrer and stirs the sample until it is dissolved. After stirring for approximately 45 min, the technician inspects the samples to see if all the polymer has dissolved and then selects the ANALYSE portion of the program to complete the analysis. The robot then removes the sample from the stirrer, places it on a titration stand, and titrates the sample with HCl using a Brinkmann 682 Titroprocessor and a 665 Dosimat. The amine end system is controlled by a PC AT compatible computer, running a BASIC program that communicates to the robot controller the major steps to perform, analyses the titration data, and calculates the final results.

### Managing change in a 'non-turnkey' environment

*Judith Pelczar-Richmond, American Cyanamid, Agricultural Research Division, Princeton, NJ*

Research is an ever-changing field. It expands and takes new direction almost daily, affecting the life of every scientist. Scientists rely on many useful tools to handle routine tasks: robots have become a powerful and versatile tool. The idea that a robot is a 'turnkey' system and not subject to change is a misconception.

Since the purchase of a Zymate II System in November 1989, the Central Sample Preparation Laboratory at American Cyanamid has undergone a number of marked changes. What are these changes? What is the impetus for these changes and how are we accommodating them? Shifting requirements demand the development and implementation of new ideas. Streamlining the routine operation to save time is one on-going goal. Addition of new PySections to the existing robotic system allows for broader distribution of chemical samples. New safety requirements, relocation of facilities, migration of data between computer databases, and quality-control issues of disposable items are some of the ever-changing issues that have been addressed.

### Automatic sample preparation for a total fluoride and tranexiamic acid quantitative analysis of toothpaste

*Shigeyuki Koike, Toshikazu Ogawa, Toyoki Sugiyama and Shigeru Hashimoto, Lion Corporation, Analytical Research Center, Tokyo, Japan*

Zymate XP systems were introduced to increase productivity of quasi-drug developments in the Lion Corporation's laboratory and quality control in its factory.

The systems prepare samples for the quantitative analysis of tranexiamic acid (TAX), sodium fluoride (NaF), and sodium monofluorophoshate ($NaFPO_3$) in toothpaste. A single XP robotic system can treat TAX, NaF and $NaFPO_3$ in 12 different toothpastes.

### Robotic automation of total nitrogen determination by chemiluminescence

*Thomas R. Smith, Gregory J. Kamla and Michael P. Kelly, Shell Development Company, Houston, TX*

The oldest continuously functioning robotic system at Shell Development Company is used for the determination of total nitrogen in organic liquids. The evolution of the robotic system over the last eight years was described. The system is based on a single robot arm that performs all aspects of the sample preparation necessary for the total nitrogen analysis. The Zymate II arm handles opening and closing the sample containers, heating of viscous sample types, transferring the sample aliquot and diluent to the dilution vial and placing the dilution vial in the auto-injector of the chemiluminescent nitrogen pyroreactor. The System V controller and pyroreactor are operated as peripheral devices by an IBM computer. All required sample information and the results of the determinations are handled by the IBM computer.

The robotic system is housed in a custom robot enclosure that conveniently integrates the robot into the laboratory environment, while increasing efficiency and reducing the amount of laboratory floor space required to perform the determination.

### Robotics automation of polyurethane test methods

*Steven E. Robbins and Michael E. Rusak, Air Products and Chemicals Inc., Allentown, PA*

Air Products is an industry leader in the production of polyurethane intermediates and additives. These are used in the production of flexible and rigid polyurethane foams typically used in the housing, furniture and automotive industries. The elastomers segment of the business supports many applications such as paper rollers, automotive parts, machine tires, and other specialized end uses.

Significant growth in polyurethane chemicals sales has generated an increasing demand for material testing support. Between 1989 and 1990, Air Products successfully developed testing systems for polyurethane foam Density and Airflow measurements (ASTM D-3574). These systems use a Zymark Zymate robot, Ames pneumatic gauges, a Mettler PM balance, and an Amscor FPI porosity instrument. Air Products developed hardware and software, with vendor supplied software, was integrated into a single test environment.

The acquisitions of the Polaroid and American Cyanamid elastomer product lines (1989 and 1991) have generated additional needs for materials testing. Prior experience in robotics automated materials testing proved to be highly effective in managing labour costs and improving both efficiency and test consistency. This expertise served as a basis for development of a more advanced robotics automation for complex sample handling approaches and the multifunctional testing requirements of elastomers. The system incorporates a Zymark XP robot, an Instron 4505 load frame, and Air Products and vendor proprietary software into an integrated work station. This system was initially applied to the elastomer Die-C Tear method (ASTM D-624).

These automated systems easily accommodate current test demands and reduce manual labour by 70–90%. They utilize less than half of their capacity, allowing significant opportunities for additional foam and elastomer test methods to be developed.

Air Products has developed intelligent software logic which allows for precision sample handling requirements and expert control of test and measurement equipment through device monitoring and correction procedures. Together, these systems are able to deliver consistency and reliability during unattended operation.

---

### BIOLOGY/BIOTECHNOLOGY

### Utilizing automation technologies in an analytical services/quality control environment: interfacing of the Tecan RSP 505 and Zymark System V microplate management system

*Joseph A. Franchetti, SmithKline Beecham Pharmaceuticals, King of Prussia, PA*

Over the past two years the author's Biopharmaceutical Analytical Services/Quality Control (AS/QC) group has analysed an average of 30 000 samples a year, of which 40% were analysed via partially automated ELISA procedures. Previously, this testing consisted of 15 hours a day (two analysts, 7·5 hours), four days a week, with a maximum of 64 samples run per day. This procedure has since become an object for total automation with the utilization of a variety of blocks to perform these tasks automatically. These blocks include: a sample request/report database, partially and totally automated assay procedures, automatic data collection and analysis, and bar-code technology for sample tracking.

Each of these blocks has the ability to function independently and also to function as a high throughput, totally automated system. The key to this high throughput is the automated assay procedure itself. This procedure utilizes two of the most advanced technologies of laboratory automation, a Tecan RSP 505 autodilutor and the Zymark System V microplate management system. The interfaced system is capable of analysing 200 samples via ELISA procedures in a 24-hour period with a single analyst's time reduced to two hours. Given its 24-hour operation, there is an enormous output which generates considerable amounts of data. This data is collected, processed, and reported automatically within our VAX mainframe.

This system has taken over a year to develop and validate, and is beginning to prove its usefulness. With the completion of the first totally automated procedure, the development of new procedures utilizing this breakthrough in technology should be less time-consuming.

Joseph Franchetti addressed applications development, problems encountered, successes observed, and the future of laboratory automation within Biopharmaceutical AS/QC and SmithKline Beecham Pharmaceuticals.

### Zymark robotic automation of antimicrobial preservative efficacy testing

*P. W. Waters, J. A. Bircher, M. A. Brinkley, M. L. Franklin and T. Bowyer, Glaxo Inc., Research Institute, Research Triangle Park, NC*

Antimicrobial Preservative Efficacy (APE) Testing assesses the effectiveness of preservative systems in various liquid drug formulations. This test is routinely performed by the Microbiology Group in the Analytical Chemistry Department at Glaxo Research Institute. The test is a time-consuming, laborious procedure that can often place a drain on laboratory resources. The authors developed a robotic system that not only automated the procedure, but reduced the risk of operator-induced contamination.

Starting with a standard Zymate II Robot Arm, Zymate II Controller, and standard Zymark PySections and software, special modifications were designed which allowed for the accurate transfer, dilution, plating, media addition, and stacking of petri dishes used to perform these microbiological assays. Custom modifications included the design and manufacture of special gripper fingers, spin stations, media dispensing stations, and petri dish storage and stacking stations.

The resulting system is user friendly and extremely flexible, allowing for testing of a variety of samples and multiple volumes simultaneously. When combined with a clean bench or portable cleanroom, the system eliminates operator-induced contamination.

### Automation for the budget-conscious laboratory: robotics back to basics

*Larry D. Sutton, Mary Kay Pappas, Werner W. Wilke and Gerald A. Ludwig, University of Iowa Hospitals and Clinics, Department of Pathology, Iowa City, IA*

Many laboratory procedures often involve repetitive, mundane and complicated tasks which, while not intellectually challenging, require a great amount of concentration over extended periods of time. The enslavement of the intellects of scientists and technicians performing these tasks is a tragic waste of human resources. With today's technology many routine laboratory procedures can be readily automated. However, as the use of laboratory robotics has grown over the past decade, so has the complexity of the systems designed for particular tasks. Such a robotics system often becomes so large and complicated that the small laboratory finds the initial investment inhibiting. Indeed these systems pose an emotional barrier to purchase because of doubts and fears pertaining to mastery of operation and maintenance. Finally, many laboratories can ill afford to dedicate multitask oriented equipment solely to one application. In the end these laboratories frequently do not opt to purchase automated systems. Yet the authors feel these laboratories should not be without these state-of-the art technologies.

The authors described the integration of a basic robotics system, purchased from Zymark Corporation, composed of a robotic arm with one syringe hand, a printer, a power and event controller and a MasterLab station, with departmental equipment in order to form a versatile automated system for: (1) the acquisition of enzyme kinetic data; (2) blood banking quality control; and (3) performing a novel, rapid technique for polymerase chain reaction experiments. The robot opens and closes sliding and hinged doors, flips switches, pipettes solutions and reagents, washes and dries the reaction cuvette, moves racks and data captures all without significant modification of department equipment. A kinetic reaction volume of 100 μl is used and parts per thousand precision has been obtained. When necessary, departmental equipment are completely accessible to individuals for manual experiments. The system demonstrates what imagination, coupled with an understanding of the robot's capabilities and software, can do for the small laboratory.

### Implementation and characterization of a NIST/NCI extraction protocol for fat soluble vitamins in human serum using the Zymark II laboratory robot

*Stephen Chesler, David Duewer, Lloyd Currie, and William MacCrehan, Chemical Science and Technology Laboratory, National Institute of Standard and Technology, Gaithersburg, MD*

A protocol for the extraction of fat soluble vitamins from human serum, developed at the National Institute of Standards and Technology (NIST) in co-ordination with the National Cancer Institute has been implemented using a Zymark II laboratory robot. This system, NCI_VITA_PREP, incorporates numerous hardware and software modifications designed to enhance: (1) pipetting of highly viscous samples and a volatile internal standard solution; (2) sample integrity; (3) error recognition and recovery; and (4) system throughput. A chemometric approach is currently being used to characterize the variability inherent in each stage of the sample preparation protocol and the effects of various environmental variables with the goal of providing highly reliable and reproducible robotic sample preparation.

An overview of the NCI_VITA_PREP system was presented, with emphasis on the steps that require hardware and software augmentation. In particular, software modifications to the pipetting, cannulating and error handling systems were described. As an alternative to the rather time-consuming and error prone, screw capping to ensure sample integrity, the concept of tube 'lidding' was explained.

The observed variability of the various steps of the extraction protocol were discussed, along with the methodology used in its determination.

### Automation of a receptor-ligand assay to achieve a high volume screen for receptor level antagonists

*S. E. Truesdell, A. L. Laborde, C. K. Marschke, J. W. Paslay, C. Y. Heckaman and J. A. Shelly, Chemical and Biological Screening, The Upjohn Company, Kalamazoo, MI*

One of the objectives of the Upjohn Company in the recent past has been to screen both fermentation broths and chemical libraries for IL-1 antagonists. In order to achieve a high volume throughput, fermentation extraction procedures and the IL-1 receptor-ligand assay were automated using laboratory robotics and multi-sample handling systems. This presentation described the development of automated procedures which allowed the assay of 2000–3000 samples per day for inhibition of IL-1 binding to YT-NCI cells. The systems that were used to accomplish this task included the Zymark Laboratory Robot, the Beckman Biomek Robotic Workstation, the Skatron Cell Harvester, and the LKB Betaplate Scintillation Counter. Microorganisms were grown in custom-made 45-well cassettes and subsequently extracted using the Zymark Laboratory Robot. Integral to the automation of this step was inclusion of a triple function 'hand', which allowed for the programming and execution of multi-task procedures. Programs for the Beckman Biomek Robotic Workstation were written to allow the instrument to handle most of the liquid transfer steps in the assay protocol. The Skatron 96-well plate harvester was used to harvest assays onto a glass fiber filtermat. Procedures were developed for the LKB Betaplate Scintillation Counter system to allow rapid sample processing and automatic data collection for transfer to data analysis programs in the Upjohn mainframe computer system. These programs wee designed with an output format that facilitated analysis and handling of screening data. The successful integration of various robotic systems made possible a rapid and efficient screen for receptor level antagonists. Moreover, the procedures implemented ensured reliability and repeatability in all aspects of the screening system.

### Solubilization: an alternative automated approach

*Shoreh N. Shabrang, Mark D. Dryzga and Bruce E. Kropscott, The Dow Chemical Company, Health and Environmental Sciences, Analytical Chemistry Laboratory, Midland, MI*

Often, manual laboratory operations must be modified to accomplish robotic automation. It is important to look for alternative ways to maintain or even improve validated procedures. The difference between success and failure is often dictated by willingness to challenge and change the established methodology.

Traditionally, in the authors Toxicology Laboratory, combustion has been used during the preparation of many biological samples for quantification of radioactivity. Samples were combusted in instruments utilizing a high temperature furnace and an oxygen atmosphere to convert radioactive carbon to carbon dioxide ($CO_2$). The $CO_2$ release was bubbled into a liquid scintillation cocktail containing a trapping solution and subsequently analysed for radioactivity. Since the oxidizer was designed for human manipulation, each sample was inserted manually while the analyst waited 2 to 4 min for complete combustion. Hundreds of samples were normally oxidized during metabolism studies, thus combustion is a very time consuming, labour intensive, and highly repetitive procedure. Automating the exact procedure would be difficult, complex and expensive.

An alternative technique to combustion uses highly caustic solvents to digest the biological samples and produces improved recovery results. In this solubilization, solvent was added to the samples and incubated at ~50 °C until samples are dissolved. The solubilized samples were mixed with liquid scintillation cocktail and appropriate solvents, then analysed for radioactivity. Solubilization is a preferable technique to combustion and is more compatible with robotic procedures. By changing the technique from combustion to solubilization and automating the solubilization procedure, the authors were able to maintain valid chemistry, process more samples, increase safety of laboratory personnel, and eliminate extensive clean-up.

The philosophy of a paradigm shift will be presented using the comparison results of the C-14 activity measurements via combustion verses robotic solubilization. The advantages of the solubilization method in general and for robotics in particular were discussed.

---

PLENARY

### Superior productivity – in the laboratory and beyond

*Francis H. Zenie, Zymark Corporation, Hopkinton, MA*

Today, the need for more productive laboratories is more compelling than ever before. As we proceed through the 1990s, intense world-wide competition and increasing regulation challenge our laboratories to ensure: more precise results, unquestioned documentation, faster turnaround and better utilization of skilled people.

Superior productivity demands the greatest economic values from our resources; people, time and capital. Not only must modern laboratories improve their internal productivity, they must enhance productivity throughout the organization.

Many leading businesses have embraced laboratory automation as a strategic foundation. They invested to gain knowledge of new technologies and experience in applying them – leading to impressive results. This presentation extends the study presented at ISLAR 1991. New, strategic insights from six leading corporations have been added to the seven in last year's study. Common elements from these real-world case histories, demonstrate powerful benefits in the laboratory and beyond.

### POSTER SESSIONS

### Zymate II dissolution robot: design modifications to improve system reliability and operation

*David D. Elks, Rick T. Davis, Marsha B. Harrawood and John A. Arnold, Burroughs Wellcome Company, Analytical Development and Quality, Assurance Laboratories, Greenville, NC 27834*

Modifications were made to the Zymate II Dissolution Robot to improve its reliability and operation. These design changes have eliminated some of the limitations of the original system and helped in the successful operation of five robotic dissolution systems at Burroughs Wellcome Co. In 1991, these systems processed over 30 000 dissolution samples for analysis.

The modifications to Zymark's robotic dissolution system include:

(1) Piston pumps to dispense dissolution media with improved accuracy, precision and speed.

(2) High capacity media storage using 40-liter heated media tanks and recirculating pumps.

(3) Stainless-steel filter tip adapters to prevent tip failure.

(4) Automated vessel covers to reduced media evaporation.

This presentation discussed the above design modifications and their impact on the operation of the systems.

### A modified PyTechnology robotic system for analysis of tablet dosage uniformity

*Ted Sadlowski, Peter F. George and Stephen Scypinski, Hoffmann-La Roche, Nutley, NJ*

To meet the demands for an increase in laboratory sample throughput without sacrificing accuracy and precision, the use of laboratory automation to analyse pharmaceutical oral dosage forms was utilized.

The flexibility of the system architecture for the Zymark PyTechnology robot allows the end user to create 'customized' PySections. Through addition of a magnetic stirring station and peristaltic pump unit as custom sections to the robotic hardware layout, tablet dosage uniformity can now be performed with a reduction in determinate error that was inherrent with the manual sample preparation procedure. The use of these customized Pysections will also allow the unit to be flexible enough to be used for the analysis of a variety of oral dosage forms (tablets, capsules and blends).

This presentation detailed the modifications performed on the robotic system and highlighted the validation of the automated sample preparation procedures used for the determination of dose uniformity of a solid oral dosage form.

### Integration of the Zymate XP and Varian 3400 gas chromatograph for polychlorinated biphenyl extraction and analysis

*Alonso Rodriquez, Donald B. Grime, Jeffrey S. Counseller and Ita Vandenbroek, Southern California Edison Company, Rosemead, CA*

The Material Testing Laboratory at Southern California Edison's Westminster facility analyses approximately 30 000 transformer oil samples for polychlorinated biphenyls (PCB) from the entire Southern California Edison system. Traditionally, this has been a highly labour-intensive procedure. Samples have to be separated into vials, placed in a specific order, documented, analysed by gas chromatograph. A computer is utilized to help in PCB identification, but many chromatograms require additional manual statistical calculations. Finally, the results are recorded in a data base for distribution to the requesting organization.

Recently, SCE decided to improve the safety and efficiency of the process by minimizing human interaction with potential carcinogens and acidified solutions. Through the use of a robot (Zymate XP) and the corresponding specific computer programming for the integration of the entire operation, these goals have been achieved.

This presentation detailed how the robot and computer systems were implemented, problems encountered and the advantages they have offered. It also demonstrated a typical analysis scenario.

### How to automate a manual solid phase extraction method

*Lynn Jordan and Richard E. Buechsenschuetz, Zymark Corporation, Hopkinton, MA*

Solid Phase Extraction (SPE) is often the method of choice for sample preparation because of the high degree specificity made possible by the wide range of packing materials and column dimensions available. SPE methods development is aided by knowing the structural characteristics of the analyte(s) of interest just as in HPLC methods development. Many laboratories perform SPE on a large number of samples. Automation of SPE sample preparation can help reduce the repetitive labour of preparing numerous samples using vacuum box technology. This paper illustrated how manual SPE methods are transferred to a BenchMate Workstation. Zymark's BenchMate Workstation controls flow rates to condition, load, rinse and elute SPE columns, which typically improves the precision as compared to vacuum box technology. The paper also briefly touched upon the concept of how to use an automated workstation for methods development and methods optimization.

### A comparative study of the extraction capabilities of various alkyl substituted silicas used in bonded phase extraction

*Philip Spraker, Edward T. Heebner and Terry L. Phillips, Worldwide Monitoring, 417A Caredean Drive, Horsham, PA 19044*

An entire series of endcapped polymeric alkyl substituted silicas ranging from $C_2$ to $C_{20}$ have been developed for use in bonded phase extraction. Experiments on these silicas have been carried out to determine analyte specificity and recovery. These results can be directly related to automated systems.

Chromatograms will be presented to graphically display variations in analyte recovery and extract cleanliness. Extraction efficiency data on a number of compounds including several barbiturates demonstrate extraction capabilities as a function of the particular alkyl group.

Generally, chromatographic cleanliness is affected by the choice of sorbent. Using the same extraction procedure, sorbents with less than eight carbon atoms per chain provide cleaner samples than the longer chain lengths. Extraneous peaks at all chain lengths are significantly reduced when the extraction column is washed with elution solvent and dried before conditioning with MeOH and water.

Hydrophobic chain configuration affects analyte recovery and cleanliness. N-butyl ($C_n4$) and isobutyl ($C_i4$) differ from tertiary butyl ($C_t4$) in selectivity. Extraction efficiency data for the analyte butabarbital using $C_n4$ as compared to $C_t4$ demonstrates this difference.

*Extraction Efficiency*
(Average of duplicate extractions)
Quantified

| *Sorbent* | *Buta-barbital* | *Amo-barbital* | *Pentobarb* | *Seco-barb* | *Dirt peaks in urine sample* |
|---|---|---|---|---|---|
| $C_n4$ | 92·9 | 96·6 | 97·7 | 97·6 | 18 271 |
| $C_t4$ | 27·6 | 73·3 | 83·8 | 98·0 | 1 336 |

### Very high volume analysis of drugs in biological fluids using a novel solid phase automated extraction system compatible with GC/NPD, GC/ECD, and GC/MS detection systems

*D. Lessard, W. Menabo and E. Valentine, Phoenix International Life Sciences, Montreal, Quebec, Canada*

A novel extraction system has been devised, which marries an automated pipetting system with multilayer cartridges. The various layers are designed to absorb or adsorb *all* components of biological fluids, including the fluid itself and any drugs in the fluid. After a residence time on the solid phase characteristic of the analyte(s), elution is achieved using an organic solvent specific to the drug class. Extracts with unusually high purity are obtained, suitable for direct injection into a GC system equipped with capillary column and very sensitive detector such as nitrogen phosphorous (NPD), electron capture (ECD) and mass spectrometer (MS). Over 100 samples per hour can be processed to the 'ready for injection' stage. Validation data from various capillary GC methods will be presented, demonstrating high sensitivity with detection limits reaching the pg/mL levels, with high recovery and excellent CVs. Use of the solid phase system, with totally automated matrix application and drug elution, combined with a short GC run time made possible by the high purity of the extract, allows analysis of over 300 plasma samples/day using 2 GCs fed by one extraction setup.

### Detailed description of a robotic system for the automated digestion of soil by the Environmental Protection Agency's Contract Laboratory Program method ILM02.0

*R. A. Stockton, Stockton Consulting Services, Richland, WA*

A robotic system for the digestion of soil by the Environmental Protection Agency's (EPA) Contract Laboratory Program (CLP) method ILM02.0 (commonly known as 3/90) was described in detail. The system is based upon the Hewlett Packard ORCA robot. Preparation of over 180 samples per day for inductively coupled plasma (ICP) and Graphite Furnace Atomic Adsorption (GFAA) analyses can be achieved with the system. Technician time required for system interactions is less than 6 hours. The system can be easily modified to perform CLP water digestions and/or EPA SW-846 Method 3050/3005/3010/3020.

Quality control results were presented. Repetitive preparation blanks, known solid laboratory control samples (CLP LCCS), and spikes demonstrated the ability of the system to properly prepare samples within the requirements of the Contract Laboratory Program.

### Laboratory automation system for standard and reagent preparation

*Lars Lindquist, Marjorie Hummel, Robert Dovi, Frank Dias and Bruce Warden, WMI-Environmental Monitoring Laboratories Inc., Geneva, IL*

Reagents and standards preparation is necessary for almost every technique used in the chemical laboratory. This is a laborious task and little effort has been made to automate this process. In 1989, the authors initiated a project to automate the preparation of 27 standards for nine various EPA approved methods and 22 reagents for six EPA approved methods. This automated system was accomplished by using a custom-built robot by Bohdan Automation. The system was justified by the following benefits:

(1) Labour savings by unattended operation.
(2) Better analyst utilization of time and instrumentation.

(3) Efficiency gains with increased sample throughput and improved turn around time.

(4) High quality of standards and reagents maintained while reducing preparation and documentation time.

(5) Gravimetric verification of all preparations.

(6) An electronic audit trail provided.

(7) Chemical handling is much safer using the robot.

The laboratory automated system is a PC menu driven, X Y Z, overhead, operational robot. The system prepares standards and reagents gravimetrically using two different size pipets and an electronic balance. The results to date show the system is equivalent to manual preparations using ASTM Class A glassware.

### Handheld computer RS232C interfacing to a peristaltic pump

*Albert Marcus Morrishow, Mount Sinai School of Medicine of the City University of New York, New York, NY*

A Hewlett-Packard Company (HP) HP48SX handheld computer was interfaced to a Scilog CP-110 peristaltic pump via RS232C. The handheld computer functions as the front-panel (control keys) for the peristaltic pump with commands (to turn the pump on and off, set the pump direction, set the pump speed, set the pump TTL output switches, and to control a dedicated 114 test tube autosampler) that are embeddable within application specific user programs. Application tasks are invoked via program softkeys. A program was derived to allow specification of a volume to be picked up and/or dispensed in a chosen time (limited by tubing and pump rates) with acceptable accuracy and precision. Up to 10 peristaltic pumps can be daisy-chained together and controlled from a single handheld computer.

### Handheld computer RS232C interfacing to a spectrophotometer

*Albert Marcus Morrishow, Mount Sinai School of Medicine of the City University of New York, New York, NY*

A Hewlett-Packard Company (HP) HP48SX handheld computer was RS232C interfaced to a Pharmacia Ultrospec III UV/Visible spectrophotometer via RS232C. The handheld computer functions as the front-panel (control keys and display) for the spectrophotometer with commands (to recalibrate, turn the deuterium lamp on and off, set absorbance or transmittance or concentration or factor modes, output a record of the wavelength, reading, and cell number, select the sample position, scan) that are embeddable within application specific user programs. Application tasks are invoked with program softkeys. Spectrophotometer acquired data are transmitted to the handheld computer, where the received ASCII text is either stored or is processed and then stored. With software available from HP, data can be sent from the handheld computer to IBM, IBM compatible personal computers, and Apple Macintosh computer workstations.

### Handheld computer RS232C interfacing to a scintillation counter

*Albert Marcus Morrishow, Mount Sinai School of Medicine of the City University of New York, New York, NY*

A Hewlett-Packard Company (HP) HP48SX handheld computer was interfaced to a Beckman 3801 scintillation counter via RS232C. The handheld computer functions as a text-buffer for the scintillation counter with data acquisition invoked via program softkeys and user prompts for sample information (i.e. number of samples). Scintillation counter acquired data are transmitted to the handheld computer, where the received ASCII text is either stored or is processed and then stored. With software available from HP, data can be sent from the handheld computer to IBM, IBM compatible personal computers, and Apple Macintosh computer workstations.

### Handheld computer RS232C interfacing to a pH meter

*Albert Marcus Morrishow, Mount Sinai School of Medicine of the City University of New York, New York, NY*

A Hewlett-Packard Company (HP) HP48SX handheld computer was interfaced to an Orion 720A pH meter via RS232C. The handheld computer functions as the front-panel (control keys and display) for the pH meter with commands (to calibrate, clear datalog, set data print mode, set date and time, select electrode channel, display list of commands, measure, change modes, display millivolts, select options menu, print, enter remote control function, check slope, set timer, enter yes and no to prompts) that are embeddable within application specific user programs. Application tasks are invoked with program softkeys. pH meter acquired data are transmitted to the handheld computer, where the received ASCII text is either stored or is processed and then stored. With software available from HP, data can be sent from the handheld computer to IBM, IBM compatible personal computers, and Apple Macintosh computer workstations.

### Automated analysis of benzodiazepines and antidepressants using Bio-Rad BZ/TCA kit

*Mohammed Mazhar, Dexter Woo and Lori Polaski, Bio-Rad Laboratories, Hercules, CA*

The Bio-Rad BZ/TCA kit was adapted for automated analysis of several benzodiazepines and antidepressants in serum. The method uses ASPEC instrument interfaced with Bio-Rad HPLC system to automate solid phase extraction and analysis.

Bio-Rad BZ/TCA kit is used for the HPLC analysis of several benzodiazepines and tricyclic antidepressants utilizing manual solid phase extraction for sample clean-up. Using ASPEC and the reagents described in Bio-Rad kit, the kit was adapted to perform the automated analysis of other antidepressants, besides benzodiazepines and tricyclics. Thus trazodone, fluxetine and norfluoxetine were also analysed by this automated method without any modification in the reagents and procedural steps described in kit.

One ml of serum sample (calibrator, control and patient) was mixed with internal standard and diluted with 1 ml of water. The vortexed and centrifuged specimen was extracted by ASPEC; eluted samples were analysed by HPLC, which was interfaced to ASPEC. Precision studies conducted at two levels of controls for all drugs gave CV less than 7%. The automated procedure met the performance criteria (precision, linearity, sensitivity) established earlier as described in the Bio-Rad BZ/TCA kit.

Correlations were conducted against the manual procedure by analysing the split sample of patient serum by both these methods. They showed good correlation.

The Bio-Rad BZ/TCA kit can be adapted for automation using ASPEC to analyse benzodiazepines and antidepressants. This automated method can be used to run large volumes of samples saving technicians time, which could be utilized for other laboratory activities. Furthermore, it allows overnight runs without any hands-on operation.

### Automated pre-column derivatization for liquid chromatography

*Kenneth Ogan and Rick Gard, Hitachi Instruments Inc., Danbury, CT*

Automation need not be restricted to addressing a complete analytical system; it can also be productively applied to selected phases or subsets of the complete system. Sample preparation and pretreatment is typically the most labour-intensive part of an analytical assay, and hence, automation of this phase of the job can be very productive. Pre-column derivatization of analytes can offer several benefits: (1) improvement of detectability by addition of an easily detected chemical tag (for example an intense chromophore); (2) selectivity enhancement in a complex sample (for example selection of only those compounds with a specific functional group); and (3) improvement of the chromatographic behaviour of a group of analytes. Automation of the derivatization process not only lessens the tedium of manual preparation, but it also offers the significant benefit of greatly improved reproducibility. A versatile HPLC autosampler was used to automate the derivatization of amino acids with orthophthalaldehye (OPA) and 9-fluorenylmethylchloroformate (FMOC), with subsequent separation by reversed-phase LC, as an example of the utility of this approach. Reproducibility was excellent, always less than 2% c.v. for peak area, and throughput very high. Application of this technique to several samples was shown.

### Automated data management system

*Craig Lacher and Diana Bye, City of Grand Forks, Grand Forks, ND*

In August 1991, an automated data management system for the authors' laboratory was initiated using existing personal computers and software. Rbase was used for DOS database software – a database was selected instead of spreadsheet because a database can hold, and selectively produce for reporting, a larger amount of data with more flexibility.

A central sample receiving area was set up using existing counters and a personal computer. The Office Assistant at the Water Plant now receives all samples, entering each sample's data on the database as each sample arrives. The Office Assistant also runs the initial analysis of pH, labels each sample, logs all data on required forms, records all sample data in the database, and places samples in the laboratory sample storage area for the chemists.

Using the reports format on the database software, automated bench sheets that are preformatted and already contain all sample data for the chemists are produced. Logsheets that previously had to be maintained in addition to the other paper work are now directly printed from the system. Completed bench sheets are given to the office staff for addition to the database. All data is verified and becomes permanent record of complete sample information. All reports required for billing, record of analysis, and notification of sample results, are produced in minutes directly from the database with all results verified.

The database is considered secure because of passwords necessary to access the database. The data can then be retrieved quickly and easily for certification officer review through on-line query of the database.

Cost savings include:

(1) Sample receiving – 4 hours per week.

(2) Bench sheet preparation – 1 hour per week.

(3) Billing – 1 hour per week.

(4) Report generation – 2 weeks per year.

Full use was made of supplies on-hand, streamlining and updating lab record keeping. This program will save 396 work hours of chemist time per year. These hours equate to $3732·83 per year after the hours added to the Office Assistant position have been subtracted. With increasing testing requirements each year, all time savings are valuable.

### Automated sample preparation for high performance liquid chromatography

*Christopher Shumate, Dan Lee, Hud Schultz and James Feenstra, Hamilton Company, Reno, NV*

Sample preparation can be extremely labour intensive and often constitutes the limiting step in HPLC analysis. Autosamplers which are integral to HPLC systems are available for nearly every commercial chromatograph. The preparation of the sample before being loaded into the autosampler has only recently been accomplished through robotic systems. Filtration and dilution are two of the most common preparation steps. Sample derivatization is often required for improved extraction, the protection of labile functionalities, improved separation, and improved detector response.

A Microlab 2200 robotic pipettor (Hamilton Company) was equipped with modular hardware to automate the common procedures associated with HPLC sample preparation. The robot automates sample extraction, filtration, dilution, internal standard addition, derivatization including heating, and injection. These procedures can be performed unattended for overnight processing of entire autosample trays or accept priority samples for preparation and direct injection. An example of these procedures was presented with timing and statistical variance highlighted.

### Implementation of an improved ELISA wash system for the Hewlett Packard microassay system

*Janice Skuse, Donna Phipps, Ron Eby, Sally Quataert and Carolyn Cimino, Praxis Biologics Inc., Rochester, NY*

A laboratory robotics microplates assay system (Hewlett Packard Microassay System) was utilized to quantitate antibody levels in serum by enzyme-linked immunosorbent assay (ELISA). All steps from preparation of sample dilutions, transfer of diluted sample to antigen coated plates, and performance of each subsequent ELISA step on assay plates including: washing of microtiter plates, addition of enzyme-labeled detection antibody, addition of substrate, and reading of absorbance values are performed using the robotics sytem. During validation it was found that variability was seen with certain antigen specific ELISAs. The absorbance readings in the wells in column 1 and row H were unacceptably high indicating an edge effect. The coefficient of variation between wells was as high as 19% when a single dilution of sample was added to all wells during the ELISA procedure. The cause of the high absorbance values in these wells was determined to be due to inadequate washing with the HP wash system. An alternative wash system employing a 403BZY microplate washer from BIO-TEK Instruments Inc. (Winooski, VT) was fully integrated into the HP microplate assay system. Validation of the BIO-TEK microplate washer was successful in that the coefficient of variation between wells was 5%, no edge effect was seen, and excellent agreement was seen between ELISA results performed either manually or using robotics. In addition to the improved quality of ELISA results, the implementation of the BIO-TEK microplate washer increased sample throughput by 33%, and freed the operator from the tedious task of wash buffer filtration which was necessary with the HP wash system.

### Robotic automation of ELISAs for genetically engineered plants

*Kurt P. Timmerman, Thomas B. Brumback, Jr, Pam Flynn, Lisa Marshall, Lyle Danielson, Manish Shelat and Lyndon Schroeder, Pioneer Hi-Bred International, Johnston, IA*

The development of genetically engineered plants requires a method to detect successfully transformed plants to track inheritance of the transformed genes through multiple generations. A system that utilizes custom automation and a Beckman Biomek 1000 Automated Laboratory Workstation to increase analytical throughput and reduce the repetitive and tedious manipulations required by manual procedures was developed by the authors.

Sample preparation is accomplished by a custom gantry robot. A paper punch is used to manually sample plant tissue which is then placed into a 1·5 ml plastic tube for storage, shipment, or immediate use. The robot adds extraction buffer, grinds the samples, centrifuges them, and transfers the supernatants from the 1·5 ml tubes into a 96 well microtiter plate at a throughput rate of 96 samples in 45 minutes.

The plate of supernatants is manually moved to a Beckman Biomek robot where the samples are first assayed for total protein concentration. Based on protein concentration, a custom program makes a variable volume transfer of each sample and dilution buffer to create a new 'standard' plate containing unform protein concentration and volumes. Using the standard plate minimizes background variation and simplifies subsequent assays. The samples in the standard plate are then assayed with a commercially available (5 Prime->Prime, Boulder, CO)NPTII ELISA kit, using the Biomek robot.

### Development of high-throughput automates by CEPH and genethon for large scale physical mapping of human genome

*P. Cohen, C. Schmit, C. Rebollo and D. Cohen, CEPH, Paris, France, and L. S. Marthe, Gonothon, France*

The speed and efficiency of constructing physical maps of human chromosomes will be significantly increased with the introduction of a new generation of high-throughput robots. For this reason the CEPH and Genethon developed three automates which aliquot DNA samples, pool libraries or display individual clones on filters. The same machines can perform a variety of other essential functions.

(1) *DNA sample distribution* – robot at CEPH used for aliquoting samples into 2 ml ampoules (capped and labelled automatically), individual wells of microplates, or the wells of agarose gels. The machine has the capacity to distribute 1500 1 ml samples into ampoules, 2500 20 μl samples onto gels or 7500 200 μl samples into the wells of microtiter plates (MTP) in 12 hours. One cycle of the machine can incorporate up to 100 MTPs, or 3024 ampoules, or 1296 sample wells on gels. The computer program allows labelling (by bar code) and sample identification to prepare complex distribution arrays. Pipette tips and tubing are washed between samples in a three-step procedure, followed by drying. As a consequence of the intensive rinsing and decontamination steps aliquoted samples have been used in PCR reactions without cross-contamination.

(2) *YAC pooling* – robot at Genethon which prepares 50 ml tubes (100 per run) containing the pools of YAC clones

(128 MTPs, 12 288 wells per run) for DNA extraction and screening, reducing the latter process by a factor of ten. One can program the desired pooling scheme. The cycle includes two stages of ultrasonic washing, and heat (300 °C) sterilizing steps. Samples and aliquots are maintained at 10 °C.

(3) *YAC filter spotting* – robot at Genethon which spots up to 1536 samples with a 96 needle applicator device onto a single nylon membrane the size of an MTP. There are two carousels each with a capacity for 300 MTPs and the robot is also used to duplicate YAC libraries. The cycle includes two stages of ultrasonic washing, a decontamination, and a heat sterilization (500 °C) stage of the 96 needle replicator device between samples. 24 membranes, 1536 samples each, are prepared in four hours.

DNA samples of CEPH families have been prepared and distributed to consortium members (H. Cann *et al.*), membranes used in the physical mapping of chromosome 21 (I. Chumakov *et al.*), and the pools used to screen the CEPH YAC library (D. Le Paslier *et al.*), which has been the source of 30 important gene localizations over the past two years. The machine's capacities and programs are evolving constantly.

### High capacity screening for renin-inhibitors

*Craig Cardella, Ermine Ciaston-Dowd, Peter Esche, Diane Thibeault and Carol Ann Homon, Boehringer Ingelheim Pharmaceuticals Inc., Ridgefield, CT*

An automated high capacity screening (HCS) assay has been established to allow rapid compound throughput in an attempt to find a novel compound which inhibits Angiotensin-1 generation from human plasma renin. The three stages of automation which comprise the Renin high capacity screening process are automated chemical dispensing, automated screening, and automated data handling.

The dispensary unit, using computer-interfaced balances, weighs compounds into bar-coded vials. The compounds are solubilized by a robotic arm and aliquoted into 96-well microtitre plates by a Hamilton AT-Plus. This instrument creates a master file of screening compounds by reading the bar-coded vials. This file is stored in the VAX database.

The Renin HCS assay of the compounds prepared by the dispensary is highly automated. A Tecan automated liquid handling system is used to perform each pipetting step of the screening assay, providing both speed and precision. The Tecan system dilutes the compounds and subsequently transfers an aliquot of the dilution into testing plates or tubes. Human plasma is added to tubes using a multi-pipetting routine which has proven to be very reliable.

After incubation of the assay tubes is complete, radioimmunoassay (RIA) analysis of the Angiotensin-1 level is performed. The Tecan prepares the standard curve followed by multi-pipetted addition of antibody and tracer to all appropriate tubes.

Upon completion of the RIA, samples are counted using a Cobra gamma counter. The RIA data generated from the Cobra is collected into a database. An automated data analysis program computes the percent inhibition for each compound screened. Finally, the analysed data is matched up with the bar-code files created by the dispensary. After verification by the analyst, an automated data entry program is used to enter compound results into the central database.

### Automated technologies for drug discovery: robotic equimolar peptide mixture synthesis

*Steven C. Banville, Janice M. Kerr, Michael A. Siani and Ronald N. Zuckerman, Chiron Corporation, 4560 Horton Street, Emeryville, CA 94608*

A fully automated peptide synthesizer has been constructed that is capable of the simultaneous synthesis of up to 36 individual peptides, and the synthesis of equimolar peptides mixtures using standard solid-phase 9-fluorenylmethoxycarbonyl (Fmoc) chemistry. The instrument consists of an array of synthesis vessels, a clevage/deprotection station, a series of solenoid valves to control liquid flow, and a Zymark robot to deliver solvents and reagents. Following peptide synthesis, resin samples are then cleaved/deprotected and extracted to provide the peptides in aqueous solution. These samples are then screened in a variety of receptor-binding assays. Details of the automated synthesis and workup procedure were presented.

### Digital imaging spectroscopy

*Mary M. Yang, KAIROS, Cambridge, MA*

Imaging spectroscopy is defined as the combination of both spatial and spectral information. An instrument has recently been developed by Professor Youvan's group at MIT (*Scientific American*, May, 1991, p. 123) that can simultaneously record the spectrum of up to 4 m pixels (or about 50 000 features). Spectra can be obtained from the blue to near infrared at 3 nm resolution. Since this spectroscopy relies on the power of image processing, it is termed Digital Imaging Spectroscopy (DIS). The effectiveness of DIS results from the use of a CCD camera, rather than a 'single-pixel' detector such as a photomultiplier. KAIROS has recently commercialized a version of this instrument for both a UNIX workstation and a PC running under Microsoft Windows. DIS can be used to determine the position and spectra of colonies directly on the surface of petri dishes, or alternatively, the spectra and concentration of substances in microtiter trays. A novel user interface to organize and display the spectral data is also provided.

### Pitfalls in setting-up a robotic sample processor (rsp) for an immunoassay laboratory, testing of performance

*I. Andersen, Novo-Nordisk A/S, Niels Steensensvej 1, DK-2820 Gentofte, Denmark*

The main pitfall in liquid handling is the lack of homogeneous dilution because of insufficient aspiration/dispensing speeds in relation to the viscosity of samples and diluents. The great sensitivity of immunoassay necessitates serial dilution of samples up to 1 M, making total homogeneity a prerequisite. Test for nonspecific adsorption of samples to the walls of tubes or microtiter-plates (mtp) have to be performed.

Another pitfall is the washing procedure of the tip if a teflon probe is used for aspiration/dispensing. The efficiency is tested by measuring the carry-over. Transfer of the final dilutions to the antibody-plate also have to be optimized. Priming of the teflon tip has to be considered, especially when diluents consist of solutions of 1–5% albumin.

Examples were given on:

(1) Testing accuracy (by weight).
(2) Testing accuracy of dilutions factors (by weight).
(3) Testing precision (by means of tartrazin solutions in mtp) by photometry.
(4) Testing carry-over (by means of tartrazin solutions in mtp) by photometry.
(5) Testing linearity (accuracy) of the photometer (by means of paranitrophenol solutions in mtp).

The precision and accuracy of pipetting equals that of the technicians using exchangeable tips.

### Automation of scintillation proximity assays for the discovery of novel cardiovascular and antiviral therapeutic agents

*Mary Jo Wildey and John Nygaard, Lederle Laboratories, Pearl River, NY*

In the pharmaceutical industry, high throughput screening is fundamental to the discovery of novel agents with therapeutic utility. The scintillation proximity assay (SPA) is a new technology being utilized by Amersham Corporation for radiometric assay development. This technology dramatically simplifies convention receptor binding and enzymatic test systems. It eliminates the time-consuming steps involved in the separation of receptor-bound from unbound ligand or substrate from product. All operations ranging from the initial addition of reagents to the final quantitation of radioactivity are conducted directly in a single tube or microtiter plate well. This new method is ideally suited to complete robotic implementation.

SPA technology has been applied at Cyanamid in the automated implementation of an assay for inhibitors of HIV protease, a specific enzyme target for the AIDS virus, and a receptor binding assay for angiotensin II (All) antagonists, which would have potential utility as antihypertensives. In SPA's, the scintillation counter detects radioisotopically labelled compounds bound to microspheres saturated with a scintillant. Thus, in the HIV protease assay, fluoromicrospheres carrying a labelled substrate are exposed to the enzyme, and counts decrease upon substrate degradation and release of the labelled product. In the All assay, the fluoromicrospheres are coated with membrane homogenates carrying All receptors, and counts increase are labelled All binds to these receptors. The Beckman Biomek SL robot adds microspheres, test samples, and enzyme or ligand to the wells of microtiter plates. After appropriate incubation times, the plates are placed directly into a Packard TopCount scintillation counter, which can process nearly 2000 samples in an overnight cycle.

